# Quetiapine monotherapy in bipolar II depression: combined data from four large, randomized studies

**DOI:** 10.1186/2194-7511-1-10

**Published:** 2013-07-04

**Authors:** Allan H Young, Joseph R Calabrese, Urban Gustafsson, Michael Berk, Susan L McElroy, Michael E Thase, Trisha Suppes, Willie Earley

**Affiliations:** Department of Psychiatry, Imperial College, London, SW7 2AZ UK; University Hospitals Case Medical Center, Case Western Reserve University, Cleveland, OH 44106 USA; Formerly AstraZeneca R&D, Södertälje, 151 85 Sweden; School of Medicine, Deakin University, Deakin, 3217 Australia; Lindner Center of HOPE, Mason, OH 45040 USA; University of Cincinnati College of Medicine, Cincinnati, OH 45229 USA; Department of Psychiatry, University of Pennsylvania School of Medicine, Western Pennsylvania, PA 19104 USA; Department of Psychiatry and Behavioral Sciences, Stanford Medical Center and VA Palo Alto Health Care System, Palo Alto, CA 94304 USA; Formerly AstraZeneca Pharmaceuticals LP, Wilmington, DE 19803 USA; Orygen Youth Health Research Centre, Centre for Youth Mental Health, Parkville, VIC, 3052 Australia; The Mental Health Research Institute of Victoria, Parkville, VIC, 3052 Australia; Department of Psychiatry, Melbourne University, Parkville, VIC, 3052 Australia; Centre for Affective Disorders, Institute of Psychiatry, King’s College, London, WC2R 2LS UK

**Keywords:** Bipolar II depression, Efficacy, Monotherapy, Quetiapine, Tolerability

## Abstract

**Background:**

Despite being present in up to 1% of the population, few controlled trials have examined the efficacy of treatments for bipolar II depression. Pooled data are presented from four placebo-controlled studies (BOLDER I [5077US/0049] and II [D1447C00135]; EMBOLDEN I [D1447C00001] and II [D1447C00134]) that evaluated the efficacy of quetiapine monotherapy for depressive episodes in patients with bipolar II disorder.

**Methods:**

All studies included an 8-week, double-blind treatment phase in which patients were randomly assigned to treatment with quetiapine 300 mg/day, quetiapine 600 mg/day, or placebo. Outcome measures included the change from baseline in MADRS total score at week 8, effect sizes, and MADRS response and remission rates.

**Results and discussion:**

Improvements in mean MADRS total scores from baseline to week 8 were significantly greater with quetiapine 300 and 600 mg/day (−15.58 [*n* = 283] and −14.88 [*n* = 289]; *p* < 0.001) compared with placebo (−11.61 [*n* = 204]). The MADRS effect sizes were 0.44 for quetiapine 300 mg/day and 0.47 for 600 mg/day (*p* < 0.001 vs placebo). Significantly higher proportions of patients receiving quetiapine, at both doses, than placebo-treated patients achieved response and remission at week 8 (*p* < 0.01). Common adverse events associated with quetiapine (both doses) included dry mouth, somnolence, sedation, dizziness, and headache. Rates of mania and hypomania were similar for quetiapine and placebo. Quetiapine monotherapy demonstrated significant efficacy compared with placebo and was generally well tolerated in the treatment of bipolar II depression.

## Background

Using *Diagnostic and Statistical Manual of Mental Disorders*, Fourth Edition (*DSM-IV*) criteria, the reported lifetime prevalence of bipolar II disorder (1.0%) is similar to the estimate for bipolar I disorder (1.1%) (Merikangas et al. 2007). Moreover, sub-threshold bipolar II disorder has been estimated to affect up to 6.7% of the general population (Angst et al. 2010). The prevalence of bipolar II disorder in clinical practice may well be underestimated. Temporally, depression usually precedes hypomania (Berk et al. 2007). The recent BRIDGE study supports earlier observations that bipolar II disorder may be misdiagnosed as major depressive disorder (Angst et al. 2012) and, in a large cohort study, at least 86% of patients whose diagnosis changed from major depressive disorder to bipolar disorder (i.e., approximately 10% of patients) had bipolar II disorder (Li et al. 2012). In another cohort study, 12.2% of patients had diagnoses changed from major depressive disorder to bipolar II disorder over a mean of 17.5 years (Fiedorowicz et al. 2011).

While the debilitating effects of bipolar I disorder are well established, the burden imposed by bipolar II disorder is gaining increasing recognition (Benazzi 2007; MacQueen and Young 2001; Valtonen et al. 2005). Among patients with bipolar II disorder, the risk of suicide, comorbidity, and role impairment is generally comparable with that of bipolar I disorder (Angst et al. 2010; Merikangas et al. 2011; Suppes and Dennehy 2002; Valtonen et al. 2005). The authors of the BRIDGE study have suggested that bipolar II disorder is even more pernicious than hitherto appreciated (Angst et al. 2011). They reported higher rates of comorbid anxiety in bipolar II disorder compared with bipolar I disorder and higher rates of anxiety disorders, eating disorders, substance use, suicide, and borderline personality comorbidity in bipolar II disorder than for unipolar disorder (Angst et al. 2011).

Depression is the prevailing phase of illness in patients with bipolar II disorder (Judd et al. 2003; Kupka et al. 2007). In a prospective study of treated patients, the percentage of time spent in depression was 37.0% for bipolar II disorder versus 36.0% for bipolar I disorder patients compared with 10% and 12.5% of the time with mania/hypomania, respectively (Kupka et al. 2007). The depression/mania ratios for patients with bipolar I and II disorder were of a similar magnitude, suggesting similar tendencies toward mood instability in the two groups. Depressive symptoms in bipolar disorder have a substantial impact on patients’ quality of life and their ability to function in everyday activities (Rosa et al. 2010; Simon et al. 2007).

There has been a paucity of evidence regarding the treatment of bipolar II depression, and, in consequence, there are few consensus recommendations for the management of these patients (Swartz and Thase 2011). Older guidelines have generally adapted recommendations for bipolar I disorder, although more recent guidelines have considered bipolar II depression separately using the extant evidence base (American Psychiatric Association 2002; Goodwin 2003, 2009; Grunze et al. 2010; Keck et al. 2004; Yatham et al. 2013). Emerging reports support the effectiveness of several therapies in the treatment of acute bipolar II depression (Cruz et al. 2010; Swartz and Thase 2011). Among these agents is quetiapine, which is the only medication approved by the United States Federal Drug Administration for monotherapy of both bipolar I and II disorder depressive episodes (Seroquel Prescribing Information 2012).

The antidepressant efficacy of quetiapine monotherapy (300 and 600 mg/day) in bipolar I and II depression was established in two pivotal, 8-week, placebo-controlled clinical trials, BOLDER (BipOLarDepRession) I and II (Calabrese et al. 2005; Thase et al. 2006). These findings were subsequently confirmed in the similarly designed EMBOLDEN (Efficacy of Monotherapy Seroquel in BipOLarDepressioN) I and II trials (McElroy et al. 2010; Young et al. 2010).

The efficacy of quetiapine in patients with bipolar II depression has been previously reported in a pooled analysis of the BOLDER I and II studies (Suppes et al. 2008). The comparable design of the BOLDER and EMBOLDEN trials facilitated a pooling of the data for patients with bipolar II depression data to increase the power of the statistical analyses. In the findings reported herein, the pooled data from all four studies are used to evaluate the efficacy of quetiapine (300 and 600 mg/day) monotherapy in patients with bipolar II depression.

## Methods

### Study design

This was a pooled analysis of a subpopulation of patients with bipolar II depression from four randomized, multicenter, double-blind, fixed-dose monotherapy studies of quetiapine versus placebo in bipolar I or II depression (BOLDER I: 5077US/0049, *N* = 542, patients with bipolar II depression: *n* = 182; BOLDER II: D1447C00135, *N* = 509, *n* = 171; EMBOLDEN I: D1447C00001, *N* = 802, *n* = 303; and EMBOLDEN II: D1447C00134, *N* = 740, *n* = 262). The studies were conducted at sites across the USA (BOLDER I and II) or at centers across the USA, Canada, and elsewhere, including sites in Europe, Asia, Turkey, Central and South America, South Africa, and Australia (EMBOLDEN I and II). All studies adhered to the current amendment of the Declaration of Helsinki and the International Conference on Harmonisation/Good Clinical Practice guidelines. Prior approvals of the study protocols and amendments were received from either a central institutional review board or a review board at the study site. Written informed consent was obtained from all patients prior to study participation.

Details on the study design have been reported previously (Calabrese et al. 2005; McElroy et al. 2010; Thase et al. 2006; Young et al. 2010). Briefly, the design of each of the four studies encompassed 8 weeks of double-blind treatment whereby patients were randomized to receive quetiapine (300 or 600 mg/day) or placebo. Additionally, the EMBOLDEN studies included the active comparators, lithium 600 to 1,800 mg/day (EMBOLDEN I) or paroxetine 20 mg/day (EMBOLDEN II) to gauge assay sensitivity (i.e., comparison vs placebo). Data from these active treatment groups have been reported previously (McElroy et al. 2010; Young et al. 2010). A continuation phase of 26 to 52 weeks’ duration also formed the design of the EMBOLDEN studies, the results for which have been reported previously (Young et al. 2012). Randomization in the studies was achieved using an interactive voice-response central randomization system, and numbers were not sequential within a site. No member of the investigational teams had access to the randomization schemes during the conduct of the studies. All treatment packaging was identical, with placebo and active tablets identical in appearance and number.

### Patient population

Eligible patients were adult outpatients aged between 18 and 65 years with a diagnosis of bipolar I or II disorder, most recent episode depressed as defined by *DSM-IV* (American Psychiatric Association 2000), with or without a rapid-cycling course (≥4 episodes to ≤8 episodes per year). Only patients with bipolar II disorder were included in the current analysis. Study participants were also required to have a Hamilton Rating Scale for Depression (Hamilton 1960) (HAM-D) total score ≥20, a HAM-D item 1 (depressed mood) score ≥2, and a Young Mania Rating Scale (Young et al. 1978) (YMRS) score ≤12.

The key exclusion criteria in the four studies were a current depressive episode >12 months’ or <4 weeks’ duration at enrollment; an Axis I disorder diagnosis other than bipolar disorder; ≥8 mood episodes in the preceding 12 months (except BOLDER I); a HAM-D Item 3 score ≥3, posing a serious suicidal or homicidal risk (as judged by the investigator), or attempted suicide within the past 6 months. In addition, a history of nonresponse to an adequate treatment period (6 weeks) with ≥2 classes of antidepressants during the current episode or previous nonresponse to the study treatments (as determined by the investigator); substance dependence (*DSM-IV*) or abuse; or a clinically relevant medical condition led to exclusion from the study.

Current antipsychotic, antidepressant, and mood-stabilizing medications were discontinued in a washout period of at least 5 to 28 days. In each study, patients were randomly assigned to once-daily quetiapine or matching placebo, which was administered orally at bedtime. An initial quetiapine dose of 50 mg/day was subsequently increased in 100-mg increments to achieve a final target dose of 300 or 600 mg/day at day 4 or day 8, respectively. Concomitant treatment with all other psychotropic drugs was prohibited during the study, with the exception of hypnotics (zolpidem tartrate up to 10 mg/day [all studies] and zaleplon up to 20 mg/day, zopiclone up to 7.5 mg/day, or chloral hydrate up to 1 g/day [EMBOLDEN I and II]) at bedtime for insomnia and lorazepam (up to 3 mg/day) for severe anxiety. Hypnotics and lorazepam were permitted for the first 3 weeks of treatment, except in the 8 h before a psychiatric assessment.

### Efficacy assessments

The primary efficacy outcome measure in all four studies was the change from baseline to week 8 in Montgomery-Åsberg Depression Rating Scale (MADRS) total score (Montgomery and Asberg 1979), and this is consequently utilized in the current *post hoc* analysis. Secondary efficacy measures included the change in MADRS individual items, MADRS response (defined as a decrease from baseline of ≥50% in MADRS total score) and remission (defined as MADRS total score of ≤12) rates, HAM-D total scores and Hamilton Rating Scale for Anxiety (Hamilton 1959) (HAM-A) total scores at week 8. Additional *post hoc* efficacy endpoints were the evaluation of effect sizes and number-needed-to-treat (NNT). Data from analyses of patient-reported outcome measures of functioning and quality of life have been reported elsewhere (Gustafsson and Fajutrao 2011). Efficacy assessments were performed at baseline and weekly (BOLDER I and II) or every 2 weeks (EMBOLDEN I and II) until week 8.

### Safety assessments

Safety and tolerability assessments included the incidence and severity of adverse events and discontinuations because of adverse events, which were recorded at each visit. Adverse events were classified according to the Medical Dictionary for Regulatory Activities (MedDRA) terminology. Additional measures were the proportion of patients experiencing treatment-emergent mania/hypomania (defined as a YMRS total score ≥16 on two consecutive assessments or at final assessment, or an adverse event report of treatment-emergent mania or hypomania) and the incidence of adverse events potentially associated with extrapyramidal symptoms (EPS; including akathisia, cogwheel rigidity, dyskinesia, dystonia, extrapyramidal disorder, freezing phenomenon, hypertonia, muscle contractions involuntary, muscle rigidity, psychomotor hyperactivity, restlessness, tardive dyskinesia, and tremor). Other safety parameters comprised weight, clinical laboratory parameters using fasting and nonfasting samples, physical examination, and vital signs.

### Statistical analyses

Data for patients with a diagnosis of bipolar II depression in the BOLDER I and II and EMBOLDEN I and II studies were pooled in order to enhance the precision of the statistical analyses. Efficacy analyses were conducted in the pooled intent-to-treat (ITT) population (patients who received at least one dose of study medication and had at least one post-baseline efficacy assessment) using last observation carried forward (LOCF) methodology. Changes from baseline in primary and secondary efficacy measures for quetiapine 300 or 600 mg/day versus placebo were evaluated using analysis of covariance (ANCOVA) with baseline score as the covariate, treatment and bipolar diagnosis strata as fixed effects, and country (EMBOLDEN I and II) or center (BOLDER I and II) as a random effect. The relationship between severity (MADRS total score at baseline) and treatment response (MADRS total score at the end of treatment) was investigated in an exploratory analysis of the ITT population by plotting the individual data and superimposing linear regression lines based on an ANCOVA with baseline score as the covariate and treatment as a fixed effect. Probability levels (*p* < 0.05, *p* < 0.01, *p* < 0.001) are provided; however, these should be viewed with caution due to the *post hoc* nature of this analysis. It should also be noted that the probability levels were not adjusted for multiplicity.

Categorical changes, such as MADRS response and remission, were analyzed with the Cochran-Mantel-Haenszel test. Effect sizes, assessed using mixed-model repeated measures (MMRM) methodology based on observed cases data, were calculated as the improvement in quetiapine score versus placebo divided by the pooled standard deviation (SD). The NNT in order to achieve response was calculated according to the formula: 1/(number of placebo responders - number of quetiapine responders); an equivalent formula calculated the NNT to achieve remission. An NNT of 1 would indicate that all patients in the quetiapine group were responders/remitters compared with none of the patients in the placebo group. Analyses of safety variables were performed on the pooled safety population (i.e., patients who received at least one dose of the study medication) and were presented descriptively.

## Results

**Figure 1:**
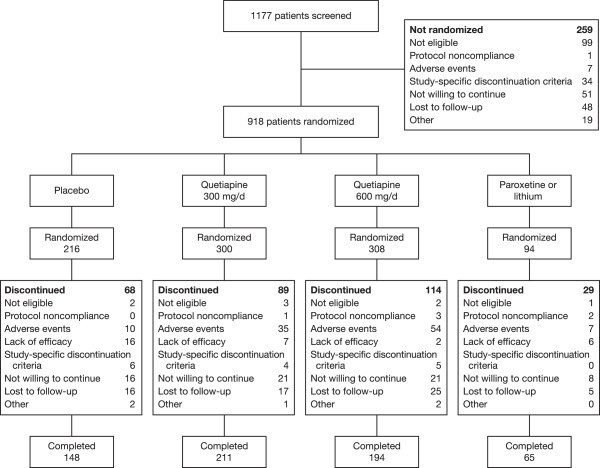
**Patient disposition.**

Patient disposition is shown in Figure 1. A total of 776 (35.2%) patients with bipolar II depression were included in the ITT population (283, 289, and 204 patients received quetiapine 300 mg/day, quetiapine 600 mg/day, or placebo, respectively). The safety population comprised 819 patients with bipolar II depression (298, 307, and 214 patients received quetiapine 300 mg/day, quetiapine 600 mg/day, or placebo, respectively), representing 35.4% of the total pooled safety population of patients with bipolar I or II depression from the four studies (*N* = 2,314).

At baseline, the demographic and clinical characteristics were similar among treatment groups (Table 1). The baseline symptom severity scores indicated a patient population with moderate to severe depression without mania symptoms.

**Table 1:** **Baseline demographic and clinical characteristics (safety population)**

	Quetiapine 300 mg/day ( ***n*** = 298)	Quetiapine 600 mg/day ( ***n*** = 307)	Placebo (***n*** = 214)
Gender,%			
Male	36.9	39.7	37.4
Female	63.1	60.3	62.6
Age, mean (years)	38.4	39.4	37.7
Weight, mean (kg)	79.7	80.1	78.9
BMI, mean	28.0	28.0	27.8
Symptom rating, mean total score			
MADRS	27.8	27.5	28.0
HAM-D	24.2	24.0	24.2
YMRS	4.5	4.5	5.0
HAM-A	19.2	18.6	19.1

### Primary efficacy outcome: Montgomery-Åsberg depression rating scale total score

**Figure 2:**
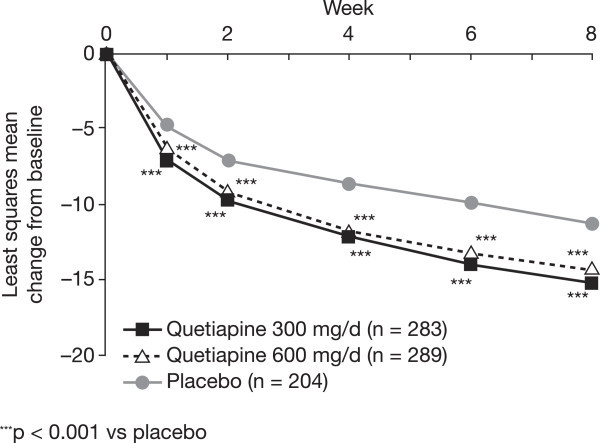
**Mean change from baseline to week 8 in MADRS total score (ITT population; LOCF).**

**Figure 3:**
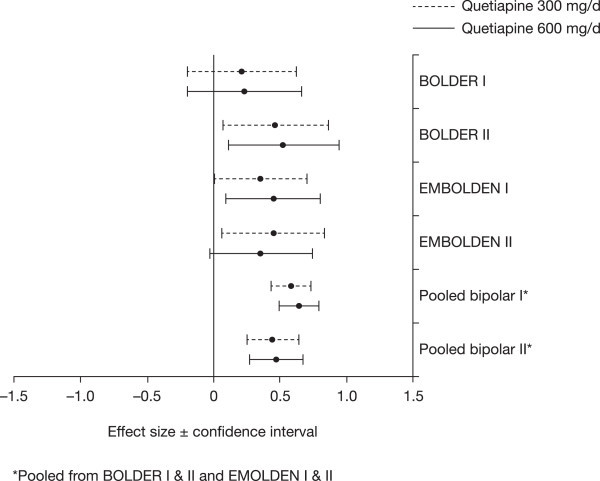
**Effect sizes of quetiapine 300 and 600 mg/day in patients with bipolar I or bipolar II disorder.**

Among patients with bipolar II disorder, treatment with quetiapine resulted in significantly greater improvements in depression symptoms compared with placebo, as measured by mean change in MADRS total score from baseline to week 8 (mean [SE] −15.58 [0.62] and −14.88 [0.62] for quetiapine 300 and 600 mg/day, respectively, vs−11.61 [0.70] for placebo; LOCF; *p* < 0.001, both doses). Significant separation from placebo was observed from week 1 for both doses of quetiapine and was maintained through week 8 (Figure 2). Effect sizes, based on MADRS scores, were moderate for both quetiapine 300 and 600 mg/day (MMRM: 0.44 and 0.47, respectively, *p* < 0.001 vs placebo; Figure 3). The effect sizes in the bipolar I population in the four trials were 0.58 for quetiapine 300 mg/day and 0.64 for quetiapine 600 mg/day (Figure 3).

**Figure 4:**
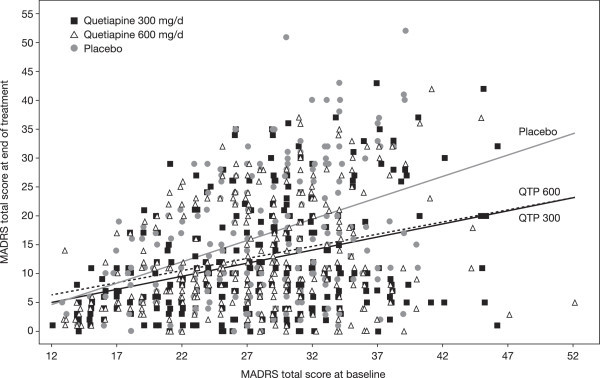
**Relationship between MADRS total score at baseline and of treatment (ITT).**

Patients with bipolar II disorder stratified by a rapid- (≥4 mood episodes per year) or nonrapid-cycling course also demonstrated significant improvements in MADRS total score at week 8. Among patients without a rapid-cycling course, MADRS total score improved significantly by a mean (SE) of 15.18 (0.67) and 14.25 (0.66) with quetiapine 300 and 600 mg/day compared with 11.58 (0.75) points for placebo (*p* < 0.001 and *p* = 0.005 for quetiapine 300 and 600 mg/day, respectively) at week 8. Significant symptomatic improvements at week 8 were also observed in patients with rapid cycling: 16.80 (1.63) and 16.92 (1.71) for quetiapine 300 and 600 mg/day versus 11.12 (1.95) for placebo (*p* = 0.011 and *p* = 0.012, respectively). In the exploratory analysis of the relationship between MADRS total score at baseline and end of treatment, there was an indication that the difference between placebo and both quetiapine doses at end of treatment increased as baseline score increased (Table 2; Figure 4). However, given the *post hoc* and exploratory nature of this analysis where the studies were not designed for this purpose, no formal inference can be made.

**Table 2:** **Relationship between MADRS total score at baseline and of treatment (ITT) (ANCOVA with baseline score as covariate and treatment as fixed effect)**

Treatment	Dependent variable	Fixed factor	Estimate	Degrees of freedom	***t*** value	***p*** value
Quetiapine 300 mg/day	MADRS at end of treatment	Intercept	−0.242	281	−0.11	0.9119
		MADRS at baseline	0.445	281	5.86	<0.0001
Quetiapine 600 mg/day	MADRS at end of treatment	Intercept	1.127	287	0.53	0.5998
		MADRS at baseline	0.423	287	5.59	<0.0001
Placebo	MADRS at end of treatment	Intercept	−4.302	202	−1.26	0.2086
		MADRS at baseline	0.741	202	6.23	<0.0001

### Secondary efficacy outcomes

#### MADRS item analyses

**Figure 5:**
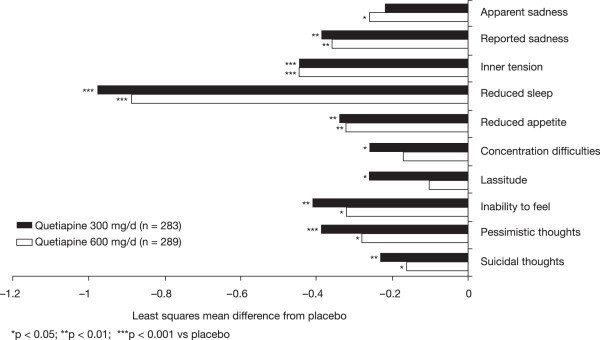
**Difference in mean change from baseline in MADRS individual item scores at week 8 (ITT population; LOCF).**

Following 8 weeks of treatment, quetiapine at doses of 300 and 600 mg/day was associated with significant improvements in the majority of the individual MADRS items when compared with placebo (*p* < 0.05 vs placebo, Figure 5), with the exceptions of apparent sadness for quetiapine 300 mg/day, and concentration difficulties and lassitude for 600 mg/day. From week 4 onward, there were significantly greater improvements in MADRS Item 10 (suicidal thoughts) scores with both doses of quetiapine (*p* < 0.05 vs placebo).

#### MADRS response and remission

By week 8, significantly higher proportions of patients treated with quetiapine 300 and 600 mg/day had achieved response and remission when compared with placebo. Among quetiapine-treated patients, 64.7% (300 mg/day) and 62.6% (600 mg/day) were classified as responders at week 8 compared with 49.0% of placebo-treated patients (*p* < 0.001 and *p* < 0.01 for 300 and 600 mg/day, respectively; ITT, LOCF). When compared with placebo (46.1%), significantly more quetiapine-treated patients met remission criteria at week 8 (65.0% and 61.9%, respectively; *p* < 0.001 vs placebo; ITT, LOCF). At week 8, NNT for response and remission were 6 and 5 for quetiapine 300 mg/day and 7 and 6 for quetiapine 600 mg/day, respectively.

#### Hamilton rating scale for depression total score

Patients who were treated with quetiapine 300 and 600 mg/day demonstrated significantly greater improvements in HAM-D total score from week 1 through week 8 compared with placebo (*p* < 0.001; ITT, LOCF). At week 8, HAM-D total scores improved by a mean (SE) of 14.00 (0.49) and 13.57 (0.49) points for quetiapine 300 and 600 mg/day, respectively, versus 10.88 (0.55) for placebo (*p* < 0.001).

#### Hamilton rating scale for anxiety total score

**Figure 6:**
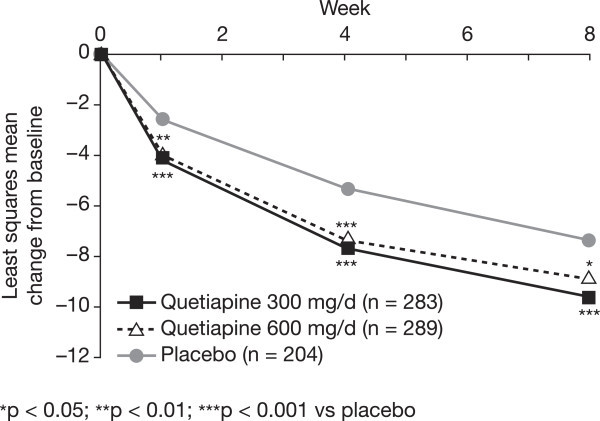
**Mean change from baseline to week 8 in HAM-A total score (ITT population; LOCF).**

Following quetiapine treatment, patients demonstrated significant improvements in symptoms of anxiety, as measured by mean change from baseline in HAM-A total score at week 8 (*p* < 0.05 both doses vs placebo; Figure 6). Significant improvements in HAM-A total score with quetiapine 300 and 600 mg/day versus placebo were observed from week 1 onward (−9.62 [*p* < 0.001] and −8.89 [*p* < 0.05] vs −7.34 at week 8, respectively; ITT, LOCF).

#### Safety

Overall rates of adverse events in the pooled safety population were 73.8%, 74.3%, and 67.8% for the quetiapine 300 mg/day, 600 mg/day, and placebo arms, respectively. As shown in Table 3, common adverse events associated with quetiapine 300 and 600 mg/day included dry mouth, somnolence, sedation, and dizziness. Adverse events potentially related to extrapyramidal symptoms were observed in 12.4%, 8.8%, and 6.1% of the quetiapine 300 mg/day, quetiapine 600 mg/day, and placebo groups, respectively. Rates of treatment-emergent mania/hypomania during acute treatment were similar among the treatment groups: 1.3%, 3.3%, and 2.3% for quetiapine 300 mg/day, 600 mg/day, and placebo arms, respectively. Rates of discontinuation because of adverse events were 12.1% for quetiapine 300 mg/day, 17.9% for quetiapine 600 mg/day, and 5.1% for placebo. Changes from baseline in weight and laboratory parameters during acute treatment are presented in Table 4. Although average weight gain was only about 1 kg greater in the two groups receiving quetiapine, the proportions of patients in both arms gaining at least 7% body weight was higher in both quetiapine arms (Table 4).

**Table 3:** **Common adverse events (≥5**% **of patients in any group; safety population)**

Adverse event, ***N*** (%)	Quetiapine 300 mg/day (***n*** = 298)	Quetiapine 600 mg/day (***n*** = 307)	Placebo (***n*** = 214)
Dry mouth	94 (31.5)	90 (29.3)	21 (9.8)
Somnolence	65 (21.8)	60 (19.5)	18 (8.4)
Sedation	60 (20.1)	59 (19.2)	12 (5.6)
Dizziness	39 (13.1)	48 (15.6)	12 (5.6)
Headache	27 (9.1)	37 (12.1)	40 (18.7)
Fatigue	26 (8.7)	30 (9.8)	14 (6.5)
Constipation	24 (8.1)	24 (7.8)	9 (4.2)
Nausea	23 (7.7)	22 (7.2)	21 (9.8)
Diarrhea	12 (4.0)	8 (2.6)	17 (7.9)
Insomnia	8 (2.7)	2 (0.7)	14 (6.5)
Upper respiratory tract infection	7 (2.3)	7 (2.3)	11 (5.1)

**Table 4:** **Weight, glucose, and lipid data (all samples; safety population)**

Mean change from randomization (SD)^a^	Quetiapine 300 mg/day (***n*** = 298)	Quetiapine 600 mg/day (***n*** = 307)	Placebo (***n*** = 214)
Total cholesterol (mg/dL)	−2.3 (2.1)	0.8 (2.0)	−3.5 (2.5)
LDL cholesterol (mg/dL)	−5.3 (1.7)	−0.3 (1.7)	−4.4 (1.9)
HDL cholesterol (mg/dL)	−0.5 (0.6)	−1.6 (0.6)	−1.0 (0.7)
Triglycerides (mg/dL)	21.4 (5.9)	14.2 (4.9)	9.0 (7.4)
Glucose (mg/dL)	3.3 (1.0)	2.8 (0.9)	1.8 (1.1)
Insulin (pmol/L)	31.0 (8.4)	24.2 (6.1)	12.5 (8.5)
Mean weight change, kg (SE)	1.0 (0.19)	1.1 (0.23)	0.0 (0.17)
Weight change ≥7%, *n* (%)	17 (6.5)	24 (9.7)	4 (2.2)

^a^Mean change from randomization to continuation phase to the end of treatment.

## Discussion

To our knowledge this pooled *post hoc* analysis of patients with bipolar II disorder experiencing an acute episode of depression represents the largest population available for evaluation to date. Quetiapine monotherapy at doses of 300 and 600 mg/day was significantly more effective than placebo at reducing the symptoms of depression as assessed by the change in MADRS total score. The improvements during quetiapine treatment were observed at week 1 and were sustained up to week 8. Significant improvements in 9 and 8 of the 10 MADRS individual items were apparent with quetiapine 300 and 600 mg/day, respectively, indicating the efficacy of quetiapine over a broad range of depressive symptoms. An exploratory analysis indicated that the difference between placebo and both doses of quetiapine at the end of treatment increased with initial severity (i.e., baseline MADRS total score). Across all analyses, the lower dose was as effective as the higher dose in patients with bipolar II disorder and tended to be associated with lower reports of adverse events.

The findings of this pooled analysis are fully consistent with those of the individual trials in which both doses of quetiapine were associated with statistically or numerically greater improvements in MADRS total score compared with placebo in the bipolar II subgroups. In the EMBOLDEN II and BOLDER II trials, quetiapine 300 and 600 mg/day resulted in significant improvements in MADRS total score from week 1 or 2 through to week 8, whereas in the EMBOLDEN I and BOLDER I trials there were no significant differences between either quetiapine dose and placebo at week 8 although there were significant differences from placebo at earlier assessments. This latter result may be explained by the relatively low power of the subanalyses in each of the trials, where the predominant number of patients had bipolar I rather than bipolar II depression.

Although these studies observed a relatively high placebo remission rate (46.1%), the remission rate for each dose of quetiapine (65.0% and 61.9% for quetiapine 300 and 600 mg/day, respectively) was nevertheless significantly higher and indicated that the benefit of active quetiapine was clinically significant. The effect sizes in the bipolar II population of the four quetiapine trials were moderate for both quetiapine 300 and 600 mg/day (0.44 and 0.47, respectively). In comparison, the effect sizes in the bipolar I population were also moderate but numerically higher (0.58 for quetiapine 300 mg/day and 0.64 for quetiapine 600 mg/day). A high placebo response rate has also been observed in other trials of agents evaluated in bipolar depression (Calabrese et al. 2008; Thase et al. 2008). Confirming the antidepressant efficacy observed on the MADRS scale, both doses of quetiapine were associated with significantly greater improvements in HAM-D total score from week 1 through week 8.

The broad therapeutic profile of quetiapine is demonstrated by the symptomatic improvements in patients with rapid cycling, who typically represent a management challenge (Bauer et al. 2008; Kupka et al. 2005; Lee et al. 2010), and may be particularly common in bipolar II (Berk and Dodd 2004; Kupka et al. 2003). A planned subanalysis of the BOLDER I study indicated that quetiapine was as effective in bipolar I or II patients with a history of rapid cycling as it was in patients with nonrapid cycling (Vieta et al. 2007). The current pooled analysis extends these observations by demonstrating significant decreases in MADRS total score at week 8 with both quetiapine doses both in patients with bipolar II depression who have rapid cycling and in those with less frequent episodes.

Comorbid anxiety is common among bipolar II patients and can have a large, adverse impact on treatment response, functioning, and quality of life (Angst et al. 2011; Gao et al. 2008; Judd et al. 2003; Otto et al. 2006). In the combined BOLDER I and II trials, quetiapine was effective in the treatment of bipolar I and II depression regardless of the severity of baseline anxiety (Lydiard et al. 2009). Patients with bipolar II depression in the BOLDER I trial did not show a statistically significant difference in HAM-A total score from placebo (Hirschfeld et al. 2006), which again may have been due to the smaller number of patients available for analysis. Quetiapine treatment resulted in significant improvements in the symptoms of anxiety in the current analysis among this larger population with bipolar II depression, the majority of whom had mild-to-moderate anxiety at baseline.

The reductions in symptom severity during quetiapine treatment in patients with bipolar II depression are accompanied by improvements in patient-reported outcomes. Pooled findings from the BOLDER and EMBOLDEN trials showed clear improvements with both doses of quetiapine in overall functioning, individual domains of social and occupational functioning, as well as quality of life (Gustafsson and Fajutrao 2011). These results are particularly promising given that a diagnosis of bipolar II disorder is associated with poor health-related quality of life even during periods of euthymia (Maina et al. 2007).

Quetiapine was tested in the four EMBOLDEN and BOLDER studies under rigorous conditions. The only other agent that has been tested under similarly robust circumstances in bipolar II depression is lamotrigine, and the data are inconsistent among studies (Swartz and Thase 2011). Among other treatments tested in bipolar II depression, the data for lithium, antidepressants (selective serotonin re-uptake inhibitors), N-acetyl cysteine, and pramipexole are suggestive of efficacy but not conclusive (Magalhaes et al. 2011; Swartz and Thase 2011). Further studies are required to establish the relative therapeutic position of these agents in the bipolar II disorder treatment armamentarium, with long-term trials being essential.

One implication of demonstrating that a medication has efficacy as an acute phase therapy is the need to document longer-term benefit for relapse prevention. The continuation phase of the EMBOLDEN studies included 231 patients with bipolar II depression who received quetiapine for 26 to 52 weeks and is one of the largest bipolar II patient populations studied in a continuation phase trial (Young et al. 2012). Quetiapine was associated with a reduced risk of any mood event or depressive relapse compared with placebo (Young et al. 2012), suggesting that it is an efficacious treatment in both the short and long term in patients with bipolar II depression.

The safety and tolerability profile of quetiapine is well established in clinical trials. In this analysis of patients with bipolar II depression, safety results during acute treatment with quetiapine were in line with observations in the individual BOLDER and EMBOLDEN patient populations (Calabrese et al. 2005; McElroy et al. 2010; Thase et al. 2006; Young et al. 2010). The adverse event profile in this patient population was qualitatively similar to findings in acute and maintenance studies of quetiapine monotherapy in patients with bipolar I or II disorder (Bowden et al. 2005; McIntyre et al. 2005; Weisler et al. 2011). However, patients with bipolar II disorder are thought to be more sensitive to side effects than bipolar I disorder patients. In the combined BOLDER I and II trials, the rate of discontinuation from quetiapine due to any adverse event was slightly higher among bipolar II disorder patients than for those with bipolar I disorder (Suppes et al. 2008).

Overall, there were changes in some glucose and lipid parameters and increases in weight with quetiapine, which were consistent with previous observations (Calabrese et al. 2005; McElroy et al. 2010; Thase et al. 2006; Young et al. 2010). The prescribing information for quetiapine recommends appropriate clinical monitoring for alterations in lipids, including blood lipid testing at the beginning of and periodically during treatment (Seroquel Prescribing Information 2012). Moreover, any patient treated with second-generation antipsychotics should be monitored for symptoms of hyperglycemia including polydipsia, polyuria, polyphagia, and weakness (Seroquel Prescribing Information 2012). When starting treatment, patients with diabetes or risk factors for diabetes should undergo blood glucose testing before and during treatment (Seroquel Prescribing Information 2012).

Treatment-emergent mania is a concern during treatment of bipolar depression with conventional antidepressants, although the extent to which it occurs in bipolar II depression remains a matter of debate (Altshuler et al. 2006; Leverich et al. 2006; Vazquez et al. 2011). In the current analyses, the rates of treatment-emergent hypomania/mania were similar for the two quetiapine doses and for placebo, indicating that quetiapine poses a minimal risk for affective switching in patients with bipolar II depression. However, the incidence of treatment-emergent mania/hypomania may have been underestimated using the definition employed in the trials (i.e., a YMRS total score ≥16 on two consecutive assessments or at final assessment or an adverse event of mania/hypomania) as patients with bipolar II disorder who exhibit frank mania may only do so briefly; moreover, patients who have a YMRS total score ≥16 on two consecutive assessments may have bipolar I disorder.

There are a number of potential limitations to this pooled analysis. An important consideration is its *post hoc* nature. Furthermore, the generalizability of the findings should be considered in the context of the inclusion and exclusion criteria of the BOLDER and EMBOLDEN trials. The patients in these studies had few significant co-morbidities and were likely to have a low risk of suicide, although this may not be the case in all patients with bipolar disorder (Perugi et al. 2013). The analysis was based on 8-week studies and gives an insight into the short-term safety and tolerability of quetiapine in this population. Longer-term information is provided by the continuation phase of the EMBOLDEN studies, which included patients with bipolar II depression who received quetiapine for up to 52 weeks (Young et al. 2012).

## Conclusions

These analyses on the largest sample of patients with bipolar II depression studied to date support the efficacy and tolerability of quetiapine 300 and 600 mg/day in treating bipolar II depression.

## Abbreviations

ANCOVA: Analysis of covariance
BOLDER: BipOLarDepRession
DSM-IV: Diagnostic and statistical manual of mental disorders, fourth edition
EMBOLDEN: Efficacy of monotherapy seroquel in BipOLarDepressioN
EPS: Extrapyramidal symptoms
HAM-A: Hamilton rating scale for anxiety
HAM-D: Hamilton rating scale for depression
ITT: Intent-to-treat
LOCF: Last observation carried forward
MADRS: Montgomery-Åsberg depression rating scale
MedDRA: Medical dictionary for regulatory activities
MMRM: Mixed-effect model repeated measure
NNT: Numbers needed to treat
YMRS: Young mania rating scale.
